# Supplementary material for: CHI3L1 enhances melanoma lung metastasis via regulation of T cell co-stimulators and CTLA-4/B7 axis
Source: Front Immunol. 2022 Dec 21;13:1056397. doi: 10.3389/fimmu.2022.1056397 (PMC9812560; doi:10.3389/fimmu.2022.1056397)
Supplement: Supplementary file 1 [file DataSheet_1.pdf]

Supplementary Table

Table S1. Sequences of RT-PCR primers used in this study

Gene	Sequence (5'to 3')	Length
Icos-S	atg aag ccg tac ttc tgc cg	20
Icos-AS	cgc att ttt aac tgc tgg aca g	22
IcosL-S	taa agt gtc cct gtt ttg tgt cc	23
IcosL-AS	att gca ccg act tca gtc tct	21
CD28-S	gtt ctt ggc tct caa ctt ctt ct	23
CD28-AS	tga ggc tga cct cgt tgc tat	21
CTLA4-S	ttt tgt agc cct gct cac tct	21
CTLA4-AS	ctg aag gtt ggg tca cct gta	21
B7-1-S	acc ccc aac ata act gag tct	21
B7-1-AS	ttc caa cca aga gaa gcg agg	21
B7-2-S	ctg gac tct acg act tca caa tg	23
B7-2-AS	agt tgg cga tca ctg aca gtt	21
Actin-S	aga ggg aaa tcg tgc gtg ac	20
Actin-AS	caa tag tga tga cct ggc cgt	21

Supplemental Figures

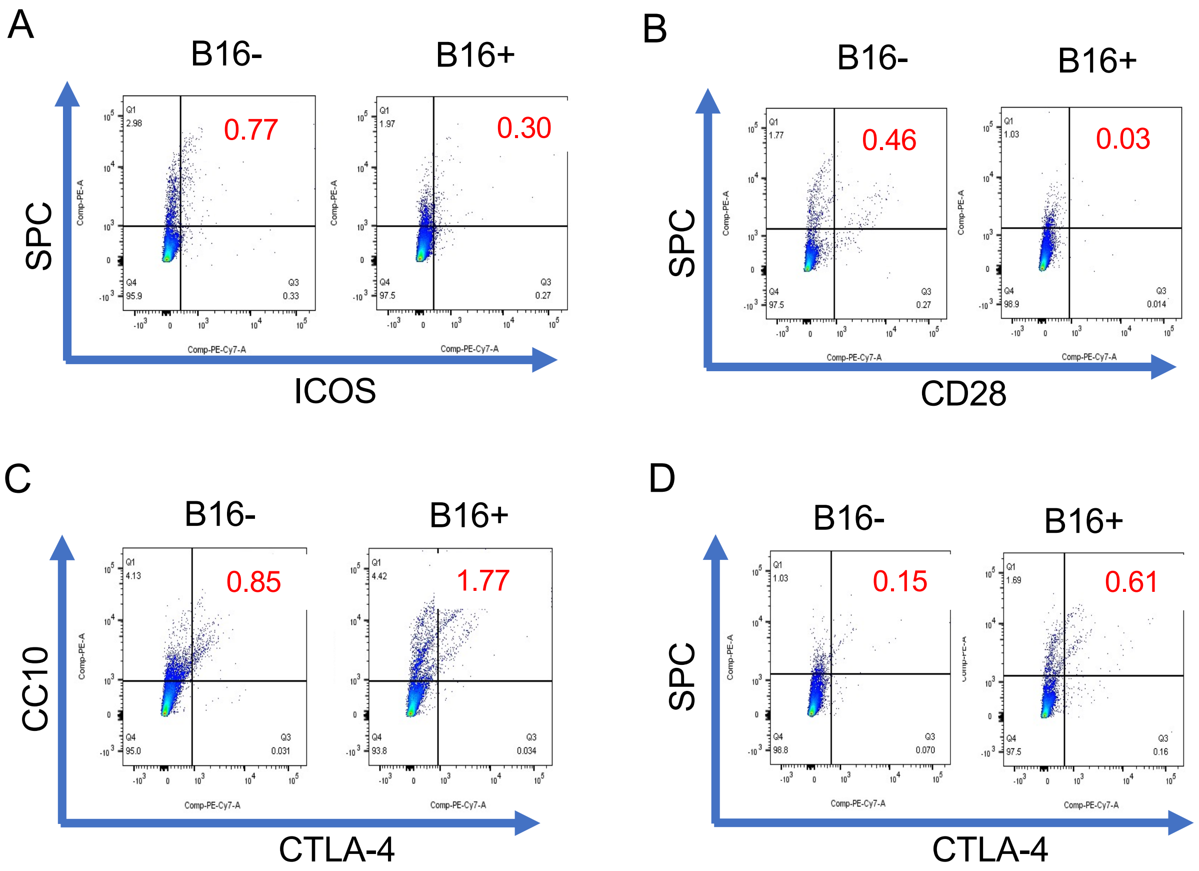

Figure S1. Expression of ICOS, CD28, CTLA4 in lung epithelial cells in melanoma lung metastasis. FACS evaluations of ICOS, CD28, and CTLA-4 expression using airway epithelial (CC10) and alveolar (SPC) epithelial cell specific markers in the lungs with melanoma (B16+) compared to vehicle (B16-) challenged mice. This is representative of a minimum of 2 similar evaluations.

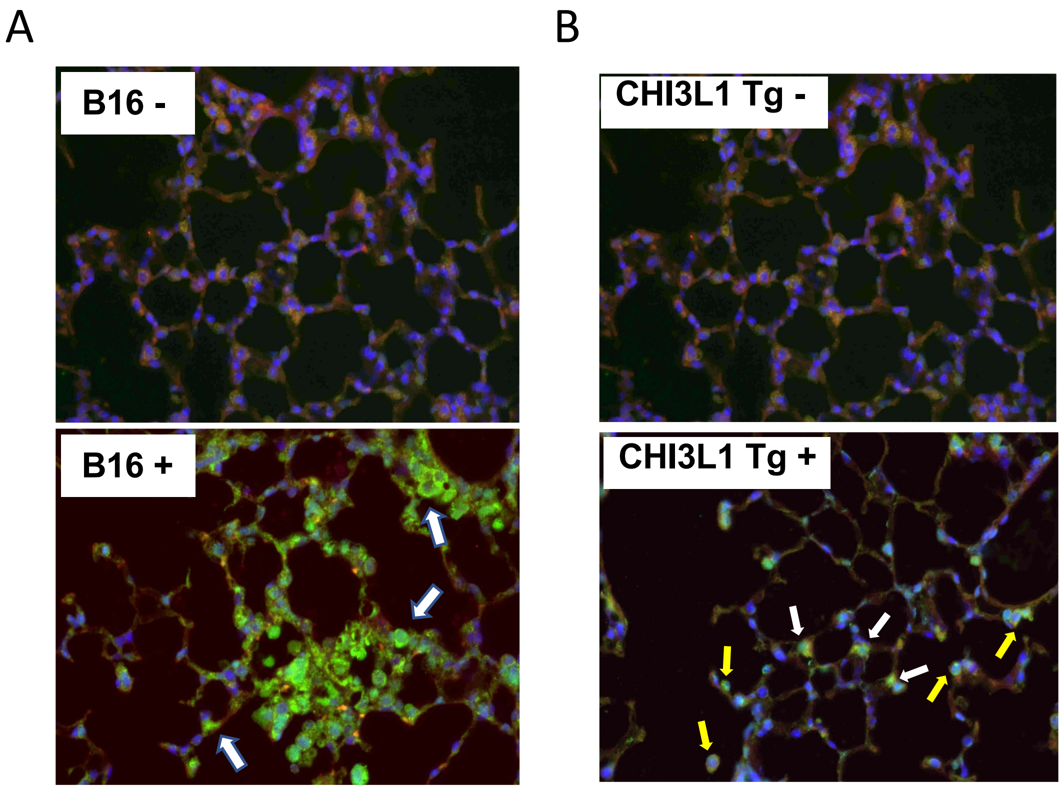

Fig. S2. CTLA-4 expression in melanoma lung metastasis and in the lungs of CHI3L1 overexpressing transgenic mice. (A) Increased expression of CTLA-4 (green, arrows) in the lungs challenged with B16 melanoma cells (B16+) compared to non-challenged ones (B16-). B) Increased expression of CTLA-4 (green) in the lungs of Chi3l1 transgenic mice (Tg+) compared to wild type (Tg-) lungs. Based on the location of the cells and morphology, the alveolar epithelial cells and macrophages/monocytes with CTLA (+) staining were identified and indicated by white and yellow arrows, respectively. Representative fluorescent immunohistochemical staining, x40 magnification.

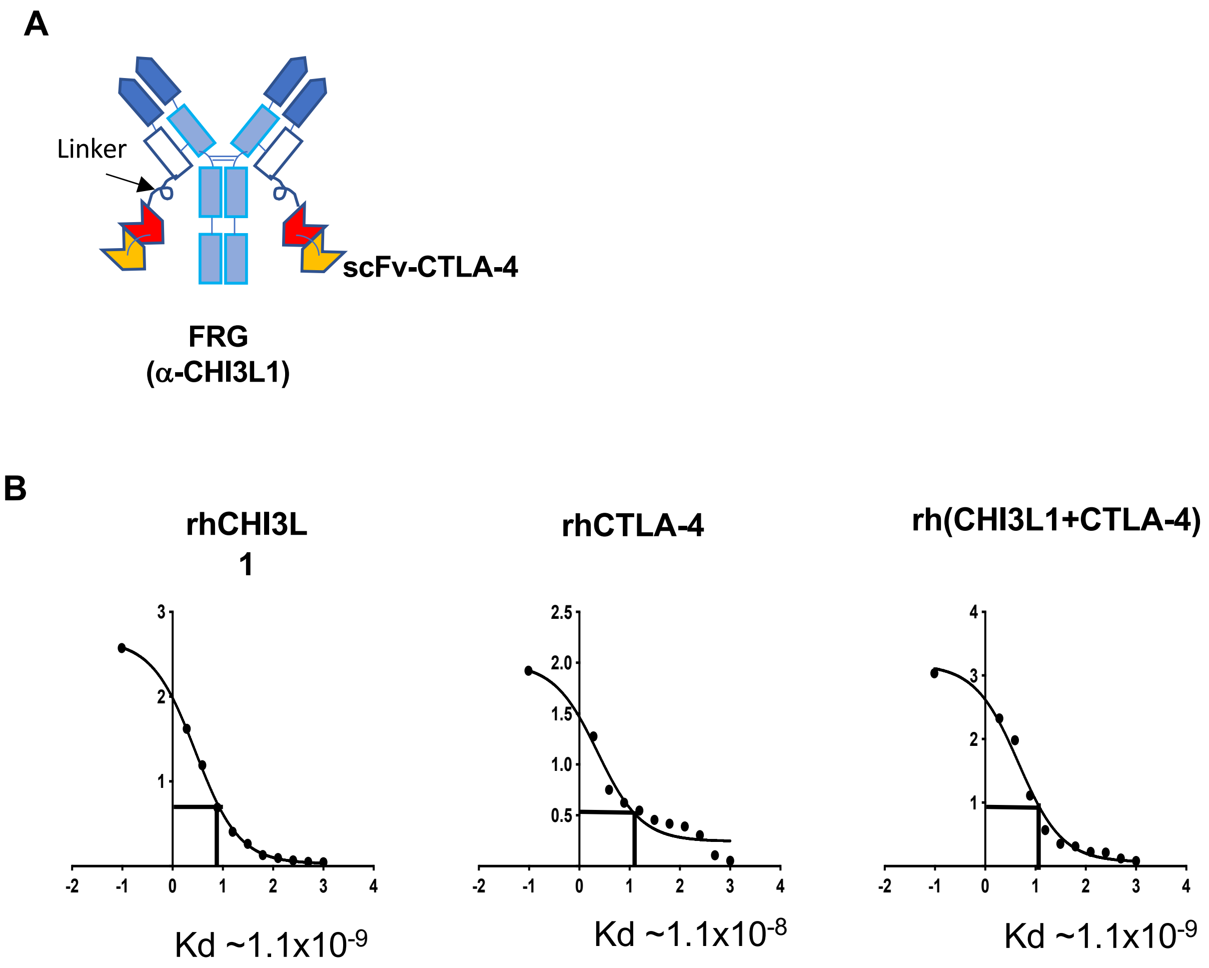

Figure S3. The structure and the binding affinity of FRGxCTLA-4 bispecific antibody. (A) Schematic illustration of the structure of the bispecific antibody FRGxCTLA4 in which anti-CTLA-4 is linked to FRG via its light chain. (B) The affinity of FRGxCTLA-4 antibody was evaluated by competitive ELISA against recombinant human (rh) Chi3l1, rhCTLA4 and mixture of rhCHI3L1 and rhCTLA-4**.**

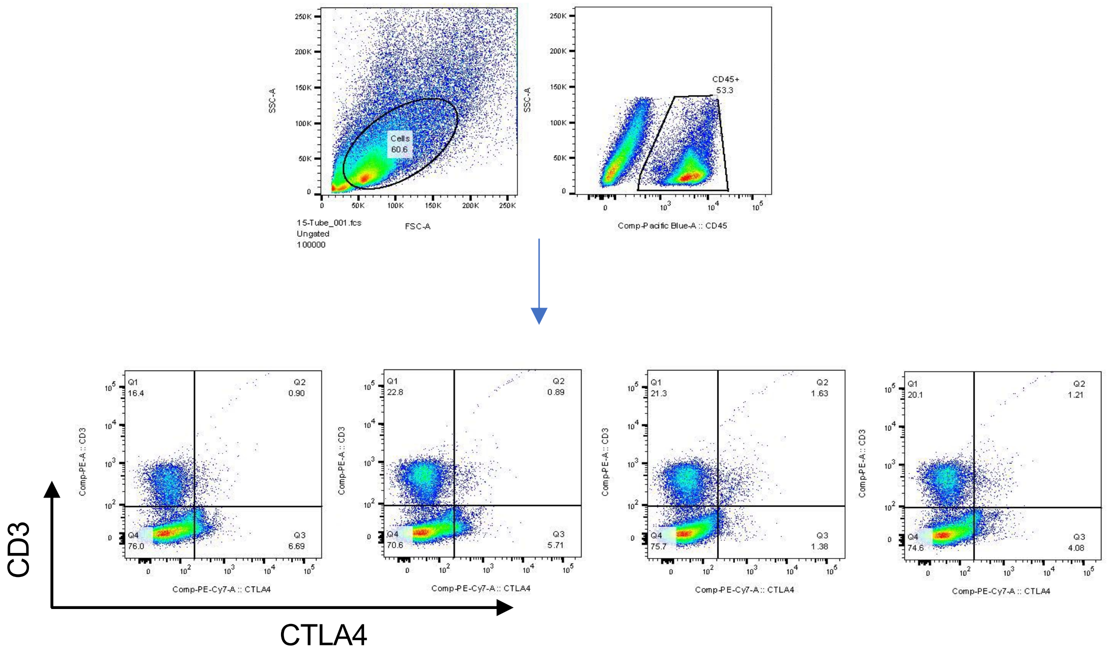

Fig. S4. Gating strategy of CD3(+)/CTLA4(+) cells in the lung. The live cells and CD45(+) cells from the lungs of individual mice were gated first then the CD3(+)/CTLA4(+) cells were counted and plotted.
